# 

**Published:** 1817-11

**Authors:** 


### CORRESPONDENCE.

The very interesting paper on Nosology, and the present State of Medicine, by a Physician, is received, and will appear, if possible, in our next Number..

Dr. Felix's remarkable Case of Plethora, successfully subdued, is thankfully received, and will shortly appear.

Communications have been received from Dr. Moulson and Mr. Simpson; as have also Dr. Bancroft's new Work on the Yellow Fever, Mr. Moore's History of Vaccination, and Sir W. Adams's Letter to the Directors of Greenwich Hospital.

Transactions of the Medico-Chirurgical Society, Vol. VIII. Part, I; and Majendie's Summary of Physiology, will be reviewed in our next. Mayo on Insanity; and Marshal on Arsenic, will be noticed as early as possible.

